# Average Type Smile in Posed Smile of Individuals Visiting Department of Orthodontics of a Tertiary Care Hospital: A Descriptive Cross-sectional Study

**DOI:** 10.31729/jnma.7072

**Published:** 2022-03-31

**Authors:** Surendra Maharjan, Anjana Rajbhandari, Resina Pradhan, Manju Bajracharya, Pushkar Manandhar, Bashu Dev Pant

**Affiliations:** 1 Department of Orthodontics, People's Dental College and Hospital, Sorakhutte, Kathmandu, Nepal

**Keywords:** *dental esthetics*, *dental photography*, *orthodontics*, *smiling*

## Abstract

**Introduction:**

New concepts and methods have been developed in orthodontics with patients' increased concern on esthetics. Among the various parameters of smile, smile type, smile arc, and smile symmetry are essential for a beautiful smile. The objective of this study was to find out the prevalence of average type in posed smiles of individuals visiting the department of orthodontics.

**Methods:**

This descriptive cross-sectional study was conducted from 1^st^ April 2021 to 30^th^ June 2021 at a tertiary care hospital. Ethical clearance was obtained from the Institutional Review Committee (Reference number: 1, CH No. 30, 2077/2078). A convenience sampling technique was used to collect a total of 413 samples of posed smile photographs. Data was collected and analysed using Microsoft Excel 2013. Point estimate at 95% Confidence Interval was calculated along with frequency and percentage.

**Results:**

Among 413 individuals, the average type of smile was found in 193 (46.73%) (42.12-51.74 at 95% Confidence Interval). Out of 87 average smile type males, most of them 51 (58.62%) had non consonant smile arcs and 47 (54.02%) had asymmetric type smiles. On the contrary, the majority of females 76 (71.70%) had a consonant arc smile while 63 (59.43%) had an asymmetric smile.

**Conclusions:**

The prevalence of the average type of smile was higher in our study as in other published literatures.

## INTRODUCTION

Smile is the effective way of communicating the emotions.^1^ It can be either enjoyment type, or posed type.^2^ Posed smile is voluntary and reproducible while spontaneous smile is involuntary and non-reproducible.^3^

In orthodontics, the treatment approach is shifting from hard tissue paradigm to soft tissue paradigm. Nowadays smiling is one of the most common causes of seeking orthodontic treatment. Thus a posed smile is of prime importance for diagnosis and treatment planning. Posed smile is evaluated in two aspects: incisal display and transverse dimension.^4^ Based on these aspects smile type was studied along with smile arc and smile symmetry. There are three types of smile: low smile, average smile, and high smile.^5,6^ Most of the studies have been done in western population and very less in Nepal.

The objective of this study was to find out the prevalence of average type smile in posed smiles of individuals visiting the department of orthodontics.

## METHODS

A descriptive cross-sectional study was conducted at Department of Orthodontics, People's Dental College and Hospital, Kathmandu, Nepal from 1^st^ April 2021 to 30^th^ June 2021. Ethical clearance was obtained from the Institutional Review Committee (Reference number: 1, CH No. 30, 2077/2078) of the same institution. A written informed consent was taken prior to taking photographs. Individuals over 18 years of age having complete permanent dentition with a pleasing soft tissue profile and competent lip condition were included. Individuals with spacing or crowding teeth, canting of the occlusal plane, gross facial asymmetries, history of orthodontic or prosthodontic treatment, lip surgery and anterior teeth not visible on posed smiles were excluded from this study. A convenience sampling technique was used. The sample size was calculated by using the formula,

n = (Z^2^ × p × q) / e^2^

  = (1.96^2^ × 0.5 × 0.5) / 0.05^2^

  = 385

Where,

n = minimum required sample size
Z = 1.96 at 95% Confidence Interval (CI)
p = prevalence taken as 50% for maximum sample size
q = 1-p
e = margin of error, 5%

The calculated sample size was 385. However, we have included 413 individuals in our study. The given number of samples were selected from the photographs of individuals visiting the department of orthodontics for consultation regarding dental treatment.

All selected individuals were photographed with posed smiles after seating them in a Natural Head Position.^7^ Photographs were taken in the same environment with the same lighting condition by using Canon 400D Digital Single Lens Reflex (DSLR) camera. The camera was fixed in position with a tripod maintaining four feet distance from the subject. The lens was positioned parallel to the face and the camera was raised to the level of the subject's lower facial third. Three smile photographs of each subject were taken and the most natural smile photograph was selected for smile analysis. The photographs were then transferred to Adobe Photoshop software, version 8.0. Then the data were collected regarding smile type, smile arc, and smile symmetry.^5^

For pretesting ten photographs were collected and study variables were analysed prior to the research study. Landmark localization and measurement using software programs was performed by primary researchers to avoid interobserver variation. For accuracy of landmark and measurement thirty photographs were selected and relocalized and re-measured by other investigators. For consistency of the procedure thirty photographs were remeasured in a one-week interval.

Data was analysed using Microsoft Excel 2013. Point estimate at 95% Confidence Interval was calculated along with frequency and percentage.

## RESULTS

Among 413 individuals, the average type of smile was found in 193 (46.73%) (42.12-51.74 at 95% Confidence Interval). It was observed that the majority of both male and females had an average type of smile, which was 87 (45.07%) female (Table 1).

**Table 1:** Demographic data of average smile individuals (n= 193).

Sex	n (%)	Age (Mean±S.D) (years)
Male	87 (45.08)	21.12±3.40
Female	106 (54.92)	19.56±1.71

While examining other related features like smile arc and smile symmetry in average smile type, it was found that, out of 87 average smile type males, most of them 51 (58.62%) had non consonant smile arc and 47 (54.02%) had asymmetric type smile. On the contrary, the majority of females 76 (71.70%) had a consonant arc smile while 63 (59.43%) had asymmetric smile (Table 2).

**Table 2:** Smile arc and smile symmetry in average smile type (n= 193).

Gender	Smile arc		Smile symmetry	
	Consonant n (%)	Non consonant n (%)	Symmetrical n (%)	Asymmetrical n (%)
Male	36	51	40	47
(n= 87)	(41.38)	(58.62)	(45.98)	(54.02)
Female	76	30	43	63
(n= 106)	(71.70)	(28.30)	(40.57)	(59.43)

## DISCUSSION

Among 413 individuals, most of them 46.73% had an average type of smile. While observing different gender, most males had average type smiles, which was the same for females. Nowadays in clinical orthodontics more focus is increasing towards the patient esthetic oriented diagnosis and treatment planning which made the smile analysis as a key analysis. Thus more focus is increasing in display zone of the patient during clinical examination which includes smile type, smile arc and smile symmetry.^8^

According to Tjan AH, et al. high smile reveals the total cervico incisal length of the maxillary anterior teeth and a contiguous band of gingiva while average smile reveals 75% to 100% of the maxillary anterior teeth and interproximal gingiva only and low smile displays less than 75% of the anterior teeth.^5^ In this study, average smile was more predominant than high and low smile type in both gender. Similar results were reported in various studies performed in different populations.^6,9–11^ Result showed that 45.08% of males have average smile type which is slightly lower than females, 54.92% which could indicate that female individuals' anterior teeth display is more during smile than that of males. The result may also be due to the presence of high lip length conditions of the males.

We observed the prevalence of relative parameters in average smile individuals i.e. smile arc and smile symmetry. Smile arc is the relationship of the curvature of the incisal edges of the maxillary incisors and canines to the curvature of the lower lip in the posed smile. In consonant smile arc the maxillary incisal edge curvature parallel to the curvature of the lower lip on smile whereas in non'consonant smile arc the maxillary incisal curvature being flatter than the curvature of the lower lip on smile.^12^ Similarly smile symmetry is the condition where the lips on each side of the smile midline symmetrical with each other.^13^

With regard to smile arc, non'consonant arc was most commonly observed in males. Fifty one (58.6%) had a non consonant smile and 36 (41.38%) had a consonant smile. In contrast females had more consonant smiles. 76 (71.70%) had a consonant smile and only 30 (28.30%) had a non consonant smile. This result was slightly different from the study done by Basnet BB, et al. in Nepali smile and Nold SL, et al. in Caucasian adults where both the gender showed non consonant smile as prominent feature.^6^’^14^ In contrast consonant smile arc was more frequent in study done in Indian and Pakistani individuals.^1,10^ During study it was noted that the smile arc was highly influenced by contour and shape of the lip. Also the attrited anterior teeth may be the contributing factor for non-consonant smiles in male.

The current result showed that more than half of the participants had an asymmetric smile, 47 (54%) in male and 63 (59.43%) in females. This result was consistent with the study of Maharjan S, et al. where majority of Nepali population had non coinciding facial and dental midline indicating asymmetry of arch.^15^ In contrast the study of Basnet BB, et al. showed only 19.47% had asymmetric exposure of smile.^6^ This result revealed that in average smile female, majority had asymmetric smile but had consonant smile which made the smile more beautiful.

The limitation of the study was that it was conducted in a single tertiary hospital of Kathmandu. More research is needed to confirm and generalise these results in a larger population. Also further study needed in different ethnic groups since Nepal resides in a diverse nature of population.

## CONCLUSIONS

The prevalence of the average type of smile was higher in our study as in other published studies. The majority of males with average smiles had non consonant and asymmetric smiles while that of females had consonant but asymmetric smiles.
